# Functional omics analyses reveal only minor effects of microRNAs on human somatic stem cell differentiation

**DOI:** 10.1038/s41598-020-60065-8

**Published:** 2020-02-24

**Authors:** Jessica Schira-Heinen, Agathe Czapla, Marion Hendricks, Andreas Kloetgen, Wasco Wruck, James Adjaye, Gesine Kögler, Hans Werner Müller, Kai Stühler, Hans-Ingo Trompeter

**Affiliations:** 1 Institute for Molecular Medicine, University Hospital Düsseldorf, Moorenstr. 5, Düsseldorf, Germany; 2 Molecular Neurobiology Laboratory, Department of Neurology, University Hospital Düsseldorf, Moorenstr. 5, 40225 Düsseldorf, Germany; 3 Institute for Transplantation Diagnostics and Cell Therapeutics, University Hospital Düsseldorf, Moorenstr. 5, Düsseldorf, Germany; 4 Department of Pediatric Oncology, Hematology and Clinical Immunology, University Hospital Düsseldorf, Moorenstr. 5, Düsseldorf, Germany; 5 Computational Biology of Infection Research, Helmholtz Center for Infection Research, Inhoffenstr. 7, 38124 Braunschweig, Germany; 6 Institute for Stem Cell Research and Regenerative Medicine, University Hospital Düsseldorf, Moorenstr. 5, Düsseldorf, Germany; 7 Biologisch-Medizinisches Forschungszentrum (BMFZ), Heinrich Heine University, Universitätsstr. 1, 40225 Düsseldorf, Germany

**Keywords:** Proteins, Nucleic acids, Proteomics, Transcriptomics, Translation

## Abstract

The contribution of microRNA-mediated posttranscriptional regulation on the final proteome in differentiating cells remains elusive. Here, we evaluated the impact of microRNAs (miRNAs) on the proteome of human umbilical cord blood-derived unrestricted somatic stem cells (USSC) during retinoic acid (RA) differentiation by a systemic approach using next generation sequencing analysing mRNA and miRNA expression and quantitative mass spectrometry-based proteome analyses. Interestingly, regulation of mRNAs and their dedicated proteins highly correlated during RA-incubation. Additionally, RA-induced USSC demonstrated a clear separation from native USSC thereby shifting from a proliferating to a metabolic phenotype. Bioinformatic integration of up- and downregulated miRNAs and proteins initially implied a strong impact of the miRNome on the XXL-USSC proteome. However, quantitative proteome analysis of the miRNA contribution on the final proteome after ectopic overexpression of downregulated miR-27a-5p and miR-221-5p or inhibition of upregulated miR-34a-5p, respectively, followed by RA-induction revealed only minor proportions of differentially abundant proteins. In addition, only small overlaps of these regulated proteins with inversely abundant proteins in non-transfected RA-treated USSC were observed. Hence, mRNA transcription rather than miRNA-mediated regulation is the driving force for protein regulation upon RA-incubation, strongly suggesting that miRNAs are fine-tuning regulators rather than active primary switches during RA-induction of USSC.

## Introduction

MicroRNAs (miRNAs) are small non-coding RNAs which primarily bind to the 3′UTR of target mRNAs in a sequence-specific manner resulting in translational repression or destabilization and degradation of the targeted mRNA^1^. In animals, primary miRNA transcripts are trimmed in a two-step process involving double strand-specific nucleases Drosha and Dicer. The mature single-stranded miRNA is then incorporated into the RNA-induced silencing complex (RISC) where functional binding to the mRNA target sequence takes place^1^. As a most important feature, miRNA-mediated regulation is characterised by a bidirectional target gene redundancy. This allows a single miRNA to target the 3′ UTRs of hundreds of mRNAs in parallel^2^. Vice versa, binding sites for several miRNAs can be found on a single 3′ UTR. In consequence of these properties, miRNAs primarily can act as network regulators being able to affect large numbers of regulatory targets in parallel^2,3^.

MiRNAs are involved in widespread biological processes throughout the entire organism also including development and disease^4^. Herein, a single miRNA can fulfil different functions in various organs or cellular environments in parallel. For example, miRNAs miR-9 and miR-124 are highly expressed in brain tissues^5^, and fulfil a variety of well described functions in regulating embryonic and adult neurogenesis, neuronal differentiation and functions^5–13^. Additionally, miR-124 fulfils functions in cells of the immune system^14^. Among other miRNAs affecting neuronal development, miR-34 was recently identified as regulator of neuronal maturation in PC12 cells by arresting cells in the G1 phase which is a prerequisite for neuronal differentiation^15^.

Systemic analyses of miRNomes and transcriptomes are established tools to identify global miRNA regulation which, for example, allows the identification of a regulatory network underlying the onset of cortical neurogenesis^16^. In addition, analysis of miRNAs and mRNAs from murine embryonic stem cells (mESC) and descendant neural stem cells led to a bioinformatic model for miRNA-based control of neuronal differentiation in mice^17^. In HeLa cells, overexpression of several miRNAs led to downregulation of hundreds of proteins albeit at a moderate level^18^, often associated with parallel decreases in mRNAs. Consequently, it has emerged in recent years that mRNA decay is the dominant regulatory miRNA effect which explains the majority (66–90%) of miRNA mediated protein repression^19,20^. However, extending the transcriptome analysis by quantitative proteome analysis is a valuable strategy to identify global, proteome-wide changes in response to miRNA regulation.

Unrestricted somatic stem cells (USSC) from hUCB are a CD45-negative subpopulation of primary cells having a multipotent differentiation potential^21^ and can be induced to pluripotency by ectopic expression of OCT4, SOX2, KLF4, and C-MYC^22^. USSC are similar to mesenchymal stem cells from bone marrow (BM-MSC), but have a specific Hox-gene expression pattern resembling that of ESC^21,23^. In addition, USSC possess longer telomeres, exhibit a significant lower senescence rate compared to BM-MSC and do not form teratoma after transplantation^21,24^. USSC have a regenerative phenotype promoting nerve regeneration after transplantation into the injured rat spinal cord^25^, at least partially, by secretion of trophic proteins and thereby influencing a multitude of relevant biological processes^26^. Using a medium comprised of retinoic acid (RA) together with growth and differentiation factors termed XXL-medium, USSC can differentiate *in vitro* into cells displaying a neuronal phenotype which have been named XXL-USSC^3,21,27^ in a time frame varying from 14–21 days. Upon incubation with XXL-medium, USSC immediately exit the cell cycle and apoptotic events lead to cell loss during ongoing XXL-treatment^27^. At the final stage of XXL-incubation, XXL-USSC have acquired a neuronal-like morphology and are characterised by expression of different neuronal markers. In addition, XXL-USSC express tyrosine hydroxylase which catalyses hydroxylation of L-tyrosine to L-DOPA, the precursor for the neurotransmitter dopamine, and release the neurotransmitter dopamine^27^. However, since USSC treated with XXL for 14 days lack action potentials they must be considered as only partially differentiated cells.

We have previously analysed the impact of miRNA expression on osteogenic and XXL-induced differentiations of USSC^3,28,29^. MiRNAs miR-26a/b and miR-29b accelerate osteogenic differentiation of USSC through targeting osteogenesis-inhibiting factors. In XXL-USSC, downregulation of 18 miRNAs primarily stemming from the miR-17-92 family was observed 14 days after induction^3^. Based on experimental target validations, these miRNAs were integrated into a regulatory network of target genes relevant for neuronal development and function^3^ and also functionally connected to the XXL induced cell cycle arrest^28^. However, these results were achieved by means of classical miRNA expression analysis as well as reporter gene-based experimental target validations followed by ectopic overexpression or inhibition of certain miRNAs. Yet, it still remains an open question how the *in vivo* regulation of miRNAs during RA-induction can affect the proteome of USSC and how the final abundance of endogenous miRNA target proteins is balanced between XXL induced initial mRNA transcription and posttranscriptional miRNA regulation.

In this study, we aim to estimate the *in vivo* impact of regulated miRNAs on the proteome of RA induced phenotypic changes of USSC by integrating tightly clocked full transcriptome and proteome data of native USSC and USSC at days 3 (3d), 7 (7d) and 14 (14d, transcriptome only) of XXL-incubation (see also Supplementary Fig. 1). Using bioinformatic target predictions combined with ectopic overexpression or inhibition of specific miRNAs we demonstrate that XXL induced transcriptional enforcement plays the dominant role in shaping protein abundance and that miRNAs play a comparatively small role, possibly acting as fine-tuners.

## Results

### Transcriptome regulation in XXL-USSC

**Figure 1:**
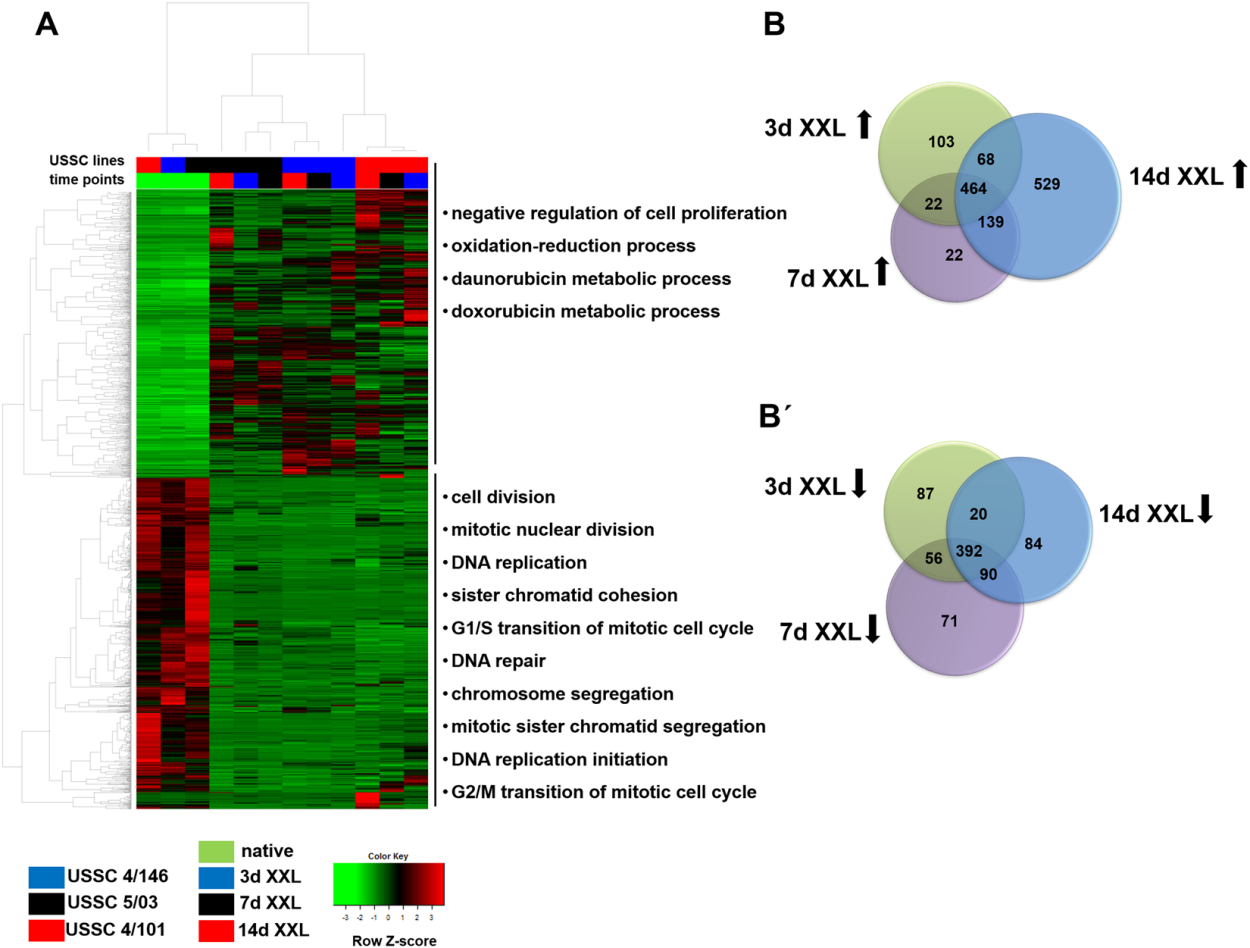
mRNA expression analysis of native and XXL-USSC. (**A**) Heat map of differentially expressed mRNAs in USSC lines 4/101, 4/146, and 5/03 (p < 0.01, q < 0.05, FC > 1.5) at time points native, 3d, 7d, 14d of XXL-incubation. USSC lines and time points are colour-coded at the top of the heat map. The heat map represents two main clusters of up- and downregulated mRNAs. Top categorical *gene ontology* enrichment annotations (q < 0.05) based on DAVID database are given for each cluster (top 10 are shown). (**B,B’**) Venn diagrams of upregulated (**B**) and downregulated (**B’**) mRNA at the respective time points of XXL incubation in comparison to native USSC.

We initially characterised the molecular signatures during XXL-medium incubation of USSC *in vitro* using an integrated approach to analyse mRNA, protein and miRNA abundances. USSC were incubated with XXL-medium as previously described^3,27,28^. XXL-induction was quality controlled by immunofluorescent staining for neurofilament as a neuronal marker and Ki-67 to proof the cell cycle exit of XXL-USSC compared to native USSC (Supplementary Fig. 2). Employing next generation sequencing, the transcriptome of USSC was analysed longitudinally (native USSC lines 4/101, 4/146, and 5/03 as well as at 3d, 7d and 14d of XXL incubation). Raw data filtering of 17,572 analysed transcripts resulted in 12,828 quantified transcripts from which 1,347 mRNAs were significantly upregulated and 800 mRNAs downregulated independent from the time point of XXL-incubation (Supplementary Tables S1 and S2). Clustering of all significantly regulated mRNAs (p < 0.01, q < 0.05, FC > 1.5) clearly demonstrated a major difference between native and XXL-USSC (Fig. 1A). Clustering revealed comparable expression patterns in native USSC over all biological replicates. In addition, cluster-specific GO-term enrichment analysis indicated that upregulated transcripts in XXL-USSC were mainly associated with metabolic processes and negative regulation of cell proliferation whereas downregulated transcripts were involved in cell cycle regulation, DNA replication and repair (Fig. 1A, Supplementary Table S3).

From all regulated genes, 464 genes were found to be upregulated and 392 genes downregulated when all time points of differentiation (3d, 7d, 14d XXL) were considered (Fig. 1B,B’). Noteworthy, 529 genes were significantly upregulated exclusively at 14d. However, enrichment analysis revealed no biological processes significantly over-represented for the latter gene set.

### Proteome regulation in XXL-USSC

**Figure 2:**
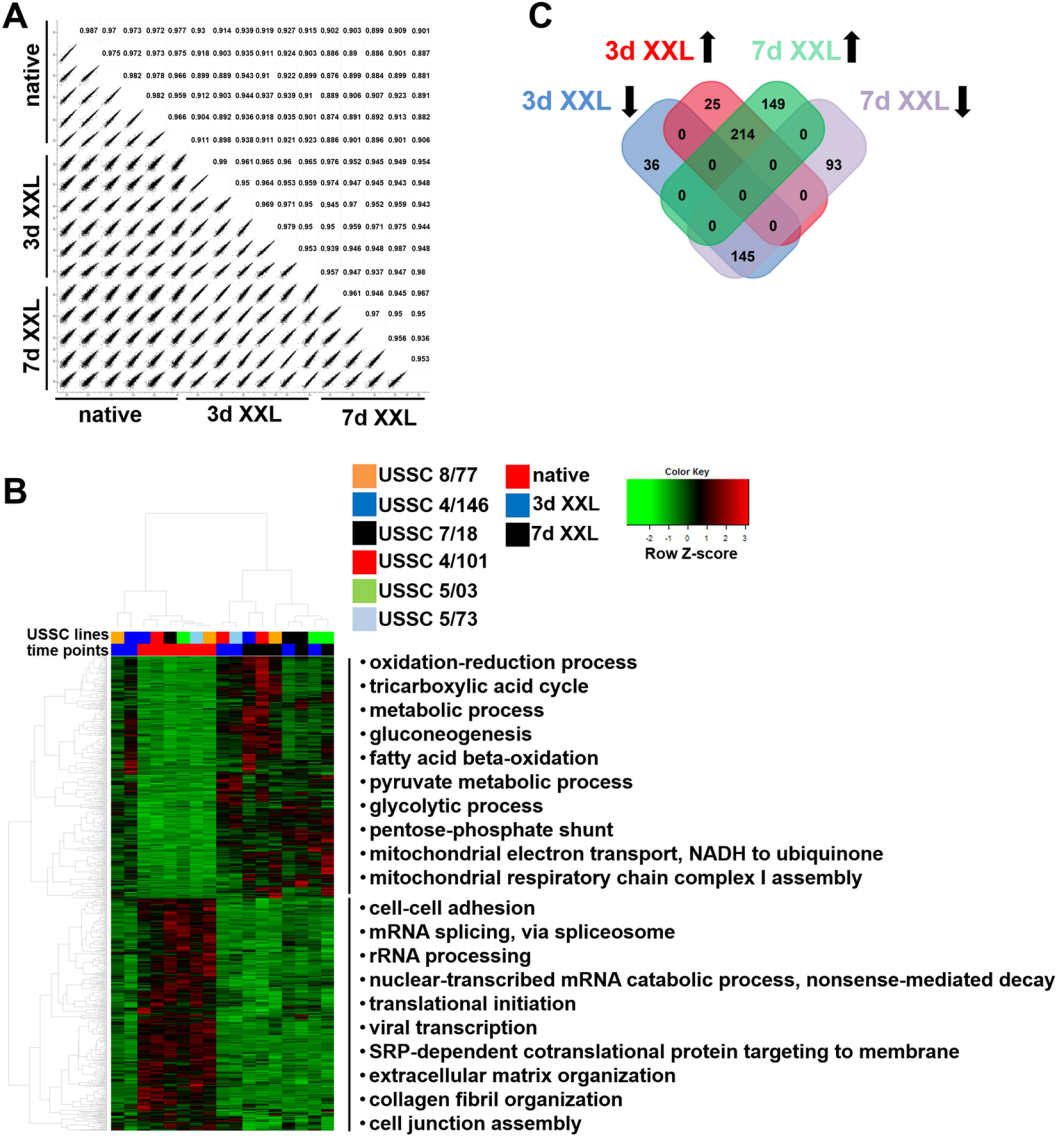
Proteome analysis of native and XXL-USSC. (**A**) Correlation plots of quantified proteins between native and XXL-treated USSC lines. Pearson’s correlations indicate a high similarity between the biological replicates. (**B**) Heat map of differentially abundant proteins in USSC lines 4/101, 4/146, 5/03, 5/73, 7/18, and 8/77 (p < 0.01, q < 0.05, FC > 1.5) at time points native, 3d, 7d, of XXL-incubation. USSC lines and time points are colour-coded at the top of the heat map. The heat map represents two main clusters of up- and downregulated proteins. Top categorical *gene ontology* enrichment annotations (q < 0.05) based on DAVID database are given (top 10 are shown). (**C**) Differentially abundant proteins at 3d and 7d XXL-USSC compared to native USSC. Black arrows denote the regulatory direction.

Quantitative label-free mass spectrometry was employed to analyse the proteome in USSC lines 4/101, 4/146, 5/03, 5/73, 7/18, and 8/77 and derived XXL-USSC. However, due to strong apoptotic events during XXL-treatment this analysis was only possible until 7d of XXL-incubation. Altogether, 1,864 proteins were included for quantitative proteome analysis (Supplementary Tables S4 and S5). Pearson’s correlation revealed high biological reproducibility at each time point (Pearson’s correlation of 0.88–0.99) (Fig. 2A). Cluster analysis demonstrated strong differences between native USSC and XXL-USSC with, however, only moderate differences between 3d and 7d XXL-incubation (Fig. 2B). Cluster-specific enrichment analysis showed that proteins with decreased abundance in differentiated USSC were assigned to biological processes associated with proliferating cells, e.g., *rRNA processing, mRNA splicing* and *translational initiation*. In XXL-USSC, proteins mainly associated with metabolic processes were increased including *gluconeogenesis* and *fatty-acid beta oxidation* as well as mitochondria associated processes (Fig. 2B, Supplementary Table S6).

In general, 388 proteins were higher abundant (p < 0.01, q < 0.05, FC > 1.5) and 274 proteins were lower abundant in XXL-USSC from which 214 proteins were higher and 145 lower abundant at both time points (3d and 7d) compared to native USSC, respectively (Fig. 2C). In addition, 149 proteins were significantly higher abundant and 93 proteins lower abundant only at 7d (Fig. 2C). Proteins once significantly induced or reduced at 3d XXL, did not show any significant inverse regulation at 7d XXL-incubation (Fig. 2C).

### Comparison of proteome and NGS based transcriptome

**Figure 3:**
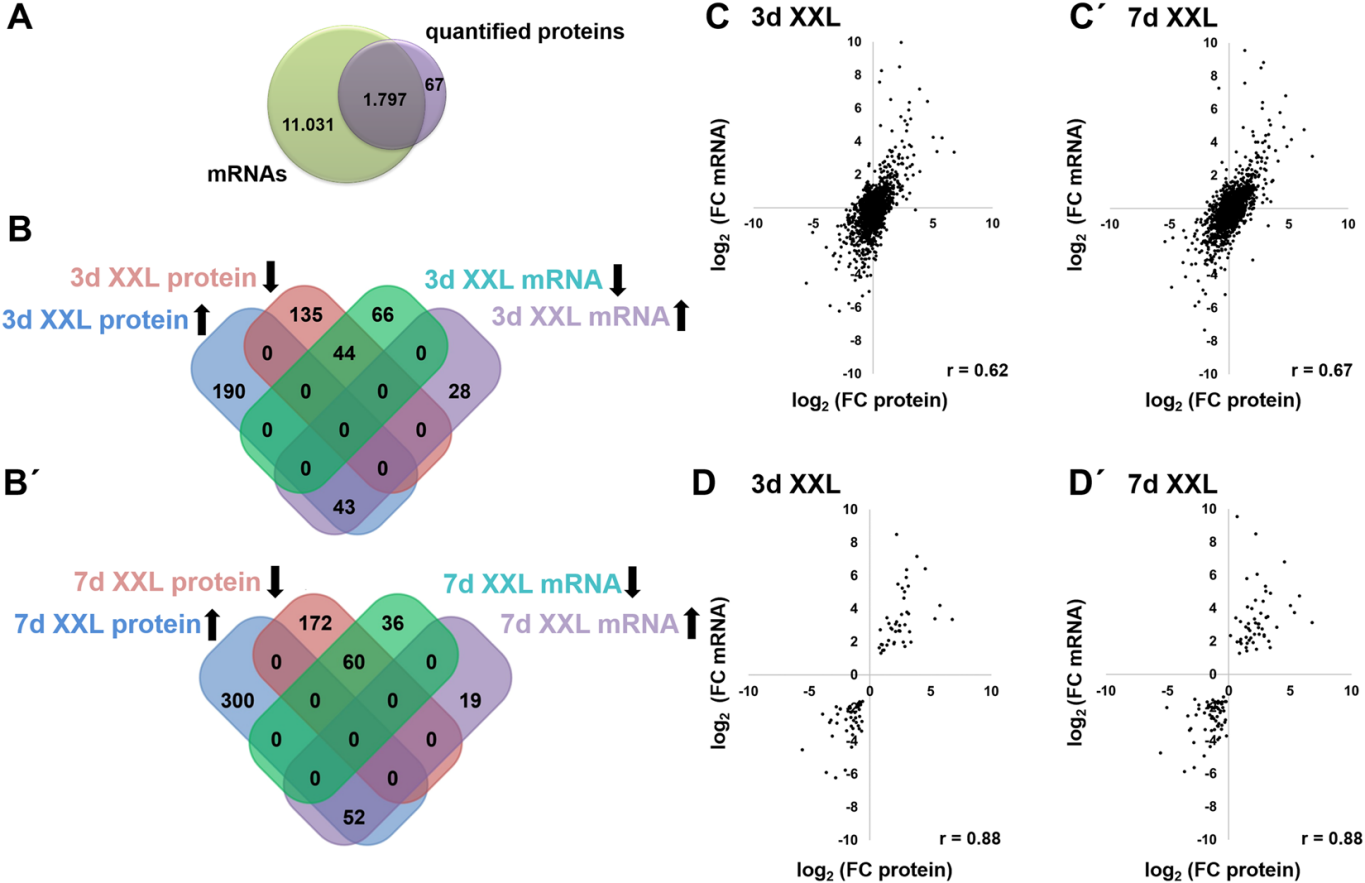
Comparison of mRNA- and proteome data in USSC and XXL-USSC. (**A**) Coverage of mRNA and protein data. From all quantified proteins, 96% were also found in the transcriptome. To exclude non-coding RNAs from the NGS data, only identified transcripts known to encode proteins were included here as revealed by UniProt database. (**B**,**B’**) Significantly regulated genes and significantly regulated corresponding proteins at 3d (**B**) and 7d (**B’**) of XXL incubation. Black arrows denote the regulatory direction. (**C**,**C’**) Correlation of mRNA- and corresponding protein regulation at 3d XXL (**C**, Pearson correlation coefficient 0.62) and 7d XXL (**C’** Pearson correlation coefficient 0.67) compared to expression levels in native USSC, respectively. (**D**,**D’**) Correlation of significantly regulated mRNAs and corresponding proteins at 3d XXL (**D**, 89 mRNAs, Pearson correlation coefficient 0.88) and 7d of XXL incubation (**D**’, 112 mRNAs, Pearson correlation coefficient 0.88) compared to expression levels in native USSC, respectively.

We next analysed whether differential abundance of mRNAs observed during XXL-incubation is also reflected in changes of corresponding proteins. Of the overall 1,864 quantified proteins, 1,797 were represented by corresponding mRNA expression (Fig. 3A). Focusing on statistically significant expression changes (p < 0.01, q < 0.05, FC > 1.5) of both, mRNAs and corresponding proteins (Fig. 3B,B’) 43 mRNAs and their related proteins were induced, and 44 mRNAs and their corresponding proteins were reduced upon 3d XXL-incubation (Fig. 3B). In 7d XXL-USSC, 52 mRNA/protein pairs were induced, and 60 mRNA/protein pairs were reduced in parallel, respectively (Fig. 3B’).

However, 28 mRNAs were induced, and 66 mRNAs were reduced at 3d XXL-incubation, without showing a parallel regulation of their related proteins. Vice versa, 190 proteins were significantly higher and 135 proteins lower abundant without significant regulation of the corresponding mRNA (Fig. 3B). At both, 3d and 7d XXL, the numbers of significantly regulated mRNAs were quite similar, however, more proteins (300) were higher abundant at 7d without regulation of corresponding transcripts (Fig. 3B’).

Independent of statistical significance most mRNAs and corresponding proteins were regulated in parallel with a Pearson’s correlation coefficient of 0.62 in 3d XXL and 0.67 in 7d XXL-USSC compared to native USSC, respectively (Fig. 3C,C’). However, the correlations were higher at both time points (0.88 each) when significantly regulated transcripts and their corresponding proteins were selected (Fig. 3D,D’). It is worth noting that only a few proteins showed an inverse regulation after XXL-induction compared to their related mRNAs. Comparison of fold changes of all 1,797 mRNAs and their corresponding proteins revealed that only 22 proteins induced by FC > 1.5 showed an inverse mRNA regulation in 3d XXL-USSC. By the same criteria, 8 proteins were reduced with the corresponding mRNAs induced at this time point (Supplementary Fig. 3A). In 7d XXL-USSC, 26 proteins were higher, and 9 proteins were lower abundant with related transcripts regulated in inverse direction (Supplementary Fig. 3B).

### MicroRNA regulation in XXL-USSC

**Figure 4:**
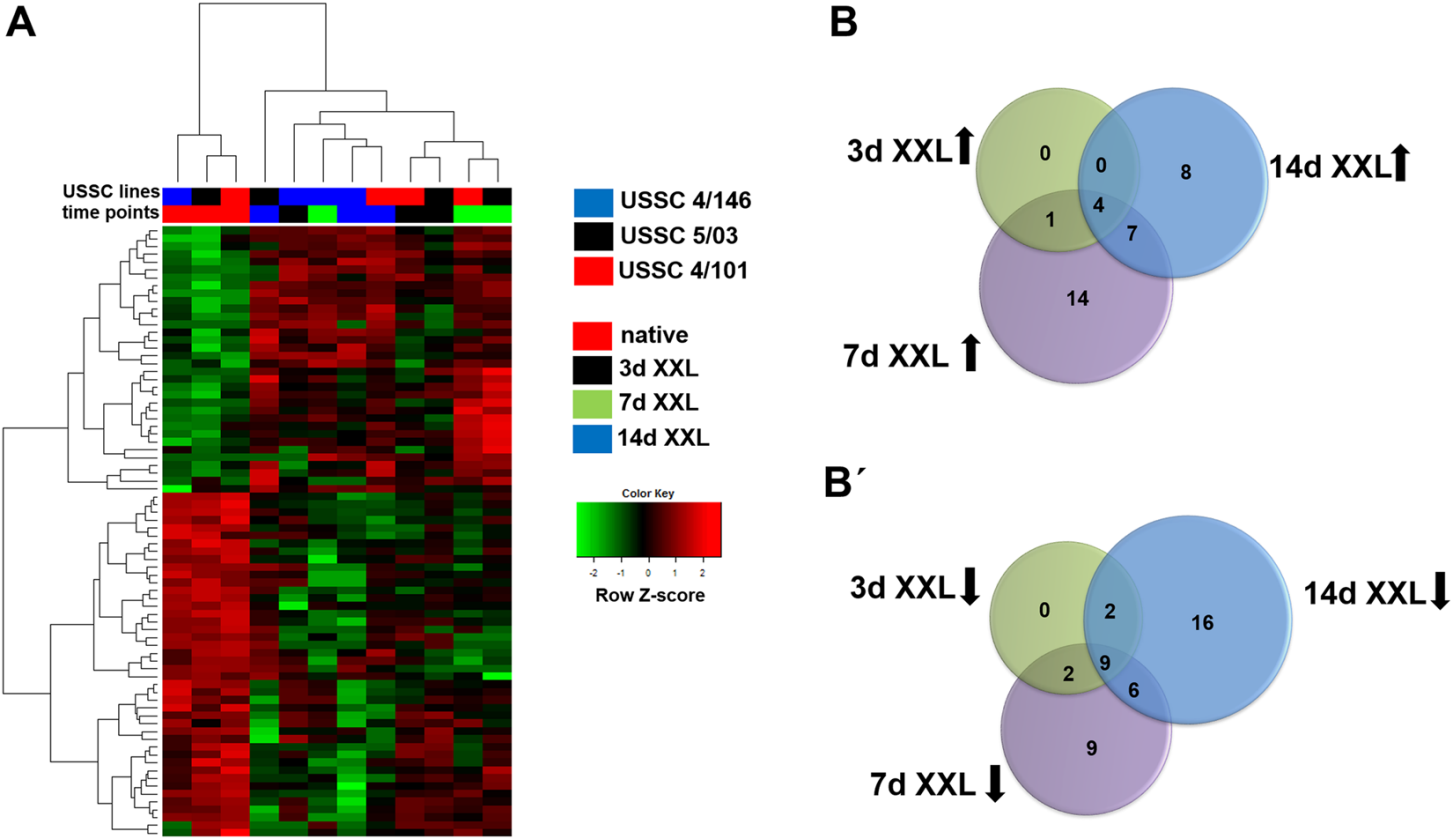
miRNA expression analysis in USSC and XXL-USSC. (**A**) Heat map of differentially expressed miRNAs in USSC lines 4/101, 4/146, and 5/03 (p < 0.01, q < 0.05, FC > 1.5) at time points native, 3d, 7d, 14d of XXL-incubation. USSC lines and time points are color-coded at the top of the heat map. (**B, B’**) Venn diagrams showing numbers of upregulated (**B**) and downregulated (**B’**) miRNAs at the respective time points of XXL incubation (3d, 7d and 14d), each compared to native untreated USSC. Black arrows denote the regulatory direction.

The aforementioned NGS dataset was analysed for miRNA expression. Herein, altogether, 750 miRNAs were quantified, 592 of which were used for fold change analyses upon filtering as described above (Supplementary Tables S7 and S8). In general, miRNA expression profiles clearly separated native USSC from XXL-USSC and miRNA expression profiles segregated further during ongoing XXL-incubation (Fig. 4A). In detail, statistical analysis revealed 5 up- and 13 downregulated miRNAs at day 3 of XXL-incubation (p < 0.01, q < 0.05, FC > 1.5) (Fig. 4B,B’). In 7d XXL-USSC, each 26 miRNAs were up- and downregulated. After 14d XXL-incubation, 19 miRNAs were up- and 33 miRNAs downregulated (Fig. 4B,B’). Among these, 8 miRNAs were up- and 16 miRNAs were downregulated exclusively at 14d XXL. Table 1 shows expression data of all miRNAs downregulated and upregulated from/to CPM > 100. Summarized, miRNomes of USSC and XXL-USSC separate from each other in a steady process from native USSC to 14d XXL-USSC.

**Table 1:** Expression pattern of significantly regulated miRNAs.

miRNA name		mean CPM				log_2_FC XXL vs. native USSC		
		native USSC	3d XXL USSC	7d XXL USSC	14d XXL USSC	3d XXL	7d XXL	14d XXL
downregulated miRNAs	hsa-miR-222-3p	18723	6440	6377	4381	−1.5	*−1.6*	*−2.1*
	hsa-miR-221-5p *	4066	566	403	594	*−2.8*	*−3.3*	*−2.8*
	hsa-miR-484	2156	530	265	1385	−2.0	*−3.0*	−0.6
	hsa-miR-24-2-5p	1096	285	167	310	*−1.9*	*−2.7*	*−1.8*
	hsa-miR-335-5p	1078	252	189	203	−2.1	*−2.5*	*−2.4*
	hsa-miR-335-3p	284	119	49	34	−1.3	−2.5	*−3.1*
	hsa-miR-27a-5p *	281	43	61	67	*−2.7*	*−2.2*	*−2.1*
	hsa-miR-431-5p	231	82	73	48	−1.5	−1.7	*−2.3*
	hsa-miR-222-5p	210	25	24	18	*−3.1*	*−3.1*	*−3.6*
	hsa-miR-138-1-3p	203	52	42	13	*−2.0*	*−2.3*	*−3.9*
	hsa-miR-17-5p	149	99	64	47	−0.6	−1.2	*−1.7*
	hsa-miR-1185-1-3p	104	37	55	22	−1.5	−0.9	*−2.2*
	hsa-miR-424-5p	101	41	21	21	−1.3	*−2.3*	*−2.3*
upregulated miRNAs	hsa-miR-146a-5p	7218	57295	60493	55211	*3.0*	*3.1*	*2.9*
	hsa-miR-191-5p	15298	29437	45496	47388	0.9	*1.6*	*1.6*
	hsa-let-7i-5p	24212	43439	76470	44284	0.8	*1.7*	0.9
	hsa-miR-34a-5p *	522	5372	5849	8076	*3.4*	*3.5*	*4.0*
	hsa-miR-23b-3p	2178	2675	3652	5662	0.3	0.7	*1.4*
	hsa-miR-132-3p	187	523	1278	706	1.5	*2.8*	*1.9*
	hsa-miR-7706	91	267	303	430	1.6	1.7	*2.2*
	hsa-miR-425-5p	203	277	1020	412	0.5	*2.3*	1.0
	hsa-miR-328-3p	61	109	231	312	0.8	*1.9*	2.3
	hsa-miR-212-5p	24	117	155	156	*2.3*	*2.7*	*2.7*
	hsa-miR-1180-3p	55	85	285	155	0.6	*2.4*	1.5
	hsa-miR-212-3p	48	171	243	153	1.8	*2.3*	1.7
	hsa-miR-182-5p	43	87	188	144	1.0	*2.1*	1.7
	hsa-miR-3605-3p	25	57	101	144	1.2	*2.0*	*2.5*
	hsa-miR-132-5p	21	57	171	60	1.5	*3.0*	1.5

Summary of miRNAs downregulated from a mean CPM value >100 (upper part of the table) and upregulated to a mean CPM value >100 at any time point of XXL incubation (lower part of the table). In XXL-USSC, 13 miRNAs were downregulated from a mean CPM value > 100 from which five miRNAs were significantly reduced at all three investigated time points of XXL incubation (hsa-miR-221-5p, hsa-miR-24-2-5p, hsa-miR-27a-5p, hsa-miR-222-5p, hsa-miR-138-1-3p). From 15 miRNAs induced in XXL-USSC, three miRNAs (hsa-miR-146a-5p, hsa-miR-34a-5p, hsa-miR-212-5p) were significantly upregulated at all investigated time points of XXL incubation. Numbers indicate the corresponding mean CPM values for the respective miRNAs at the four time points. Expression changes are shown as log_2_FC values at the right side. Log_2_ fold values in *italics* indicate significant expression changes. The miRNAs with * were used for transfection experiments.

### Bioinformatic integration of expression data

To initially estimate the contribution of miRNAs regulated in USSC upon XXL-induction we performed an extensive bioinformatic miRNA target gene prediction survey employing all 12 algorithms implemented in miRWalk 2.0^30,31^ with p-value <0.01 and otherwise default settings. From the miRNAs presented in Table 1, we selected candidate miRNAs which were significantly regulated (p < 0.01, q < 0.05, FC > 1.5) at all three analysed time points during XXL-incubation. Thus from the group of downregulated miRNAs, we chose hsa-miR-221-5p, hsa-miR-24-2-5p, hsa-miR-27a-5p, hsa-miR-222-5p, and hsa-miR-138-1-3p. From the upregulated miRNAs, hsa-miR-146a-5p, hsa-miR-34a-5p, and hsa-miR-212-5p matched the criteria. Target predictions resulted in average 14,116 non-redundant putative target genes for each miRNA, ranging from 3,673 targets for hsa-miR-24-2-5p up to 18,205 putative targets for hsa-miR-27a-5p (Supplementary Tables S9 and S10) compared to the aforementioned 12,828 identified transcripts in USSC (Supplementary Tables S1–S3).

**Figure 5:**
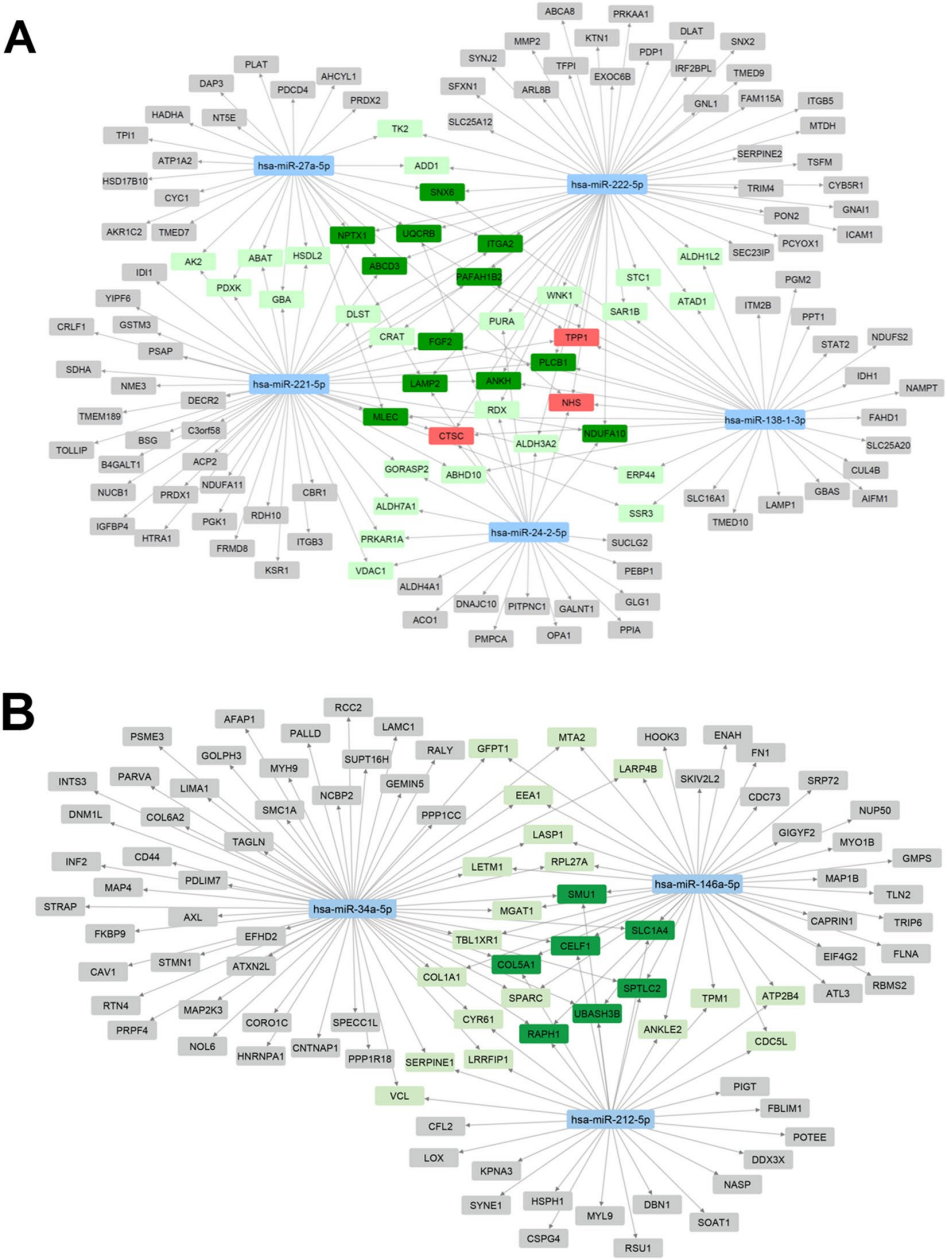
Virtual networks of regulated miRNAs and predicted target proteins inversely regulated in XXL-USSC. Networks were generated from miRNAs significantly regulated (p < 0.01, q < 0.05, FC > 1.5) at all time points of XXL-incubation and proteins significantly regulated in inverse direction (3d and/or 7d XXL) that are predicted as targets of the respective miRNA by at least 5 algorithms. Blue boxes: miRNAs. Proteins are colour-coded for the number of predicting miRNAs: grey, 1 miRNA; light green, 2 miRNAs; dark green 3 miRNAs; red, 4 miRNAs. (**A**) Network including downregulated miRNAs and higher abundant predicted target proteins. (**B**) Network including upregulated miRNAs and lower abundant predicted target proteins.

To estimate the effect of regulated miRNAs on the proteome of XXL-USSC we next cross-matched the predicted target genes for these regulated miRNAs with proteins inversely regulated in XXL-USSC at day 3 and/or day 7. To create networks of regulated miRNAs and predicted inversely regulated target proteins we limited the number of putative targets to those being predicted in parallel by at least five out of twelve algorithms used by miRWalk 2.0. The resulting networks for down- and upregulated miRNAs are given in Fig. 5. The full crossmatches of the regulated miRNAs and unlimited number of predicted target proteins inversely regulated at 3d and 7d XXL-induction are given in Supplementary Tables S9 and S10.

For both, down- and upregulated miRNAs the predicted networks (Fig. 5) initially imply a strong impact of the regulated miRNome on the proteome of XXL-USSC, since many proteins are predicted targets of more than a single miRNA. When based on all putative targets without limitations in numbers of algorithms, the crossmatches (Supplementary Tables S9 and S10) revealed that for all miRNAs analysed, only a small fraction of regulated proteins were not predicted as targets for the regulated miRNAs.

### Proteome analysis upon ectopic overexpression and inhibition of microRNAs

The bioinformatic data integration points to networks of regulated miRNAs and inversely regulated proteins (Fig. 5). However, the large amounts of putative targets generated by the unfiltered predictions (Supplementary Tables S9 and S10) challenge the reliability of such bioinformatic analyses since they exceed the number of identified transcripts in USSC. In addition, the quite similar regulation of mRNAs and proteins outlined in Fig. 3 raised questions regarding the real *in vivo* contribution of miRNAs to the final protein abundance in XXL-USSC. Therefore, we tested the real impact of candidate miRNAs on proteome formation by their ectopic overexpression or inhibition followed by proteome analysis of transfected XXL-USSC.

Three representative candidates from the miRNAs used for the bioinformatic integration (see also Table 1) were selected. Herein, both regulatory directions as well as absolute expression strengths of the miRNAs in native USSC were taken into account. Hsa-miR-221-5p is the most strongly expressed miRNA in native USSC (mean CPM: 4066) that is significantly reduced at all analysed time points. Hsa-miR-27a-5p represents a group of miRNAs far weaker (CPM < 1000) expressed in USSC (mean CPM: 281) but still significantly reduced at all time points. Hsa-miR-34a-5p is the most strongly upregulated miRNA in XXL-USSC.

**Figure 6:**
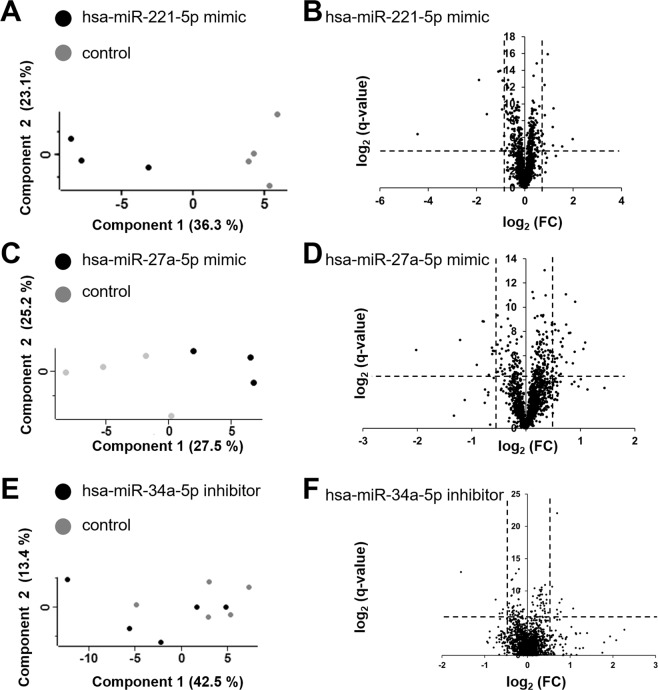
Proteome analysis of miRNA mimic/inhibitor transfected XXL-USSC at 3d XXL. Principle component analyses (PCA) and Volcano Plots of 3d XXL-USSC transfected with hsa-mir-221-5p mimic (**A,B**), hsa-mir-27a-5p mimic (**C,D**) and hsa-mir-34a-5p inhibitor (**E,F**) in comparison to n.t. siRNA transfected USSC (unspecific control). (**A,C,E**) PCAs showing separation of mimic/inhibitor transfected 3d XXL-USSC from control transfected 3d XXL-USSC. (**B,D,F**) Corresponding Volcano plots displaying differentially abundant proteins from transfected 3d XXL-USSC compared to the unspecific control.

**Figure 7:**
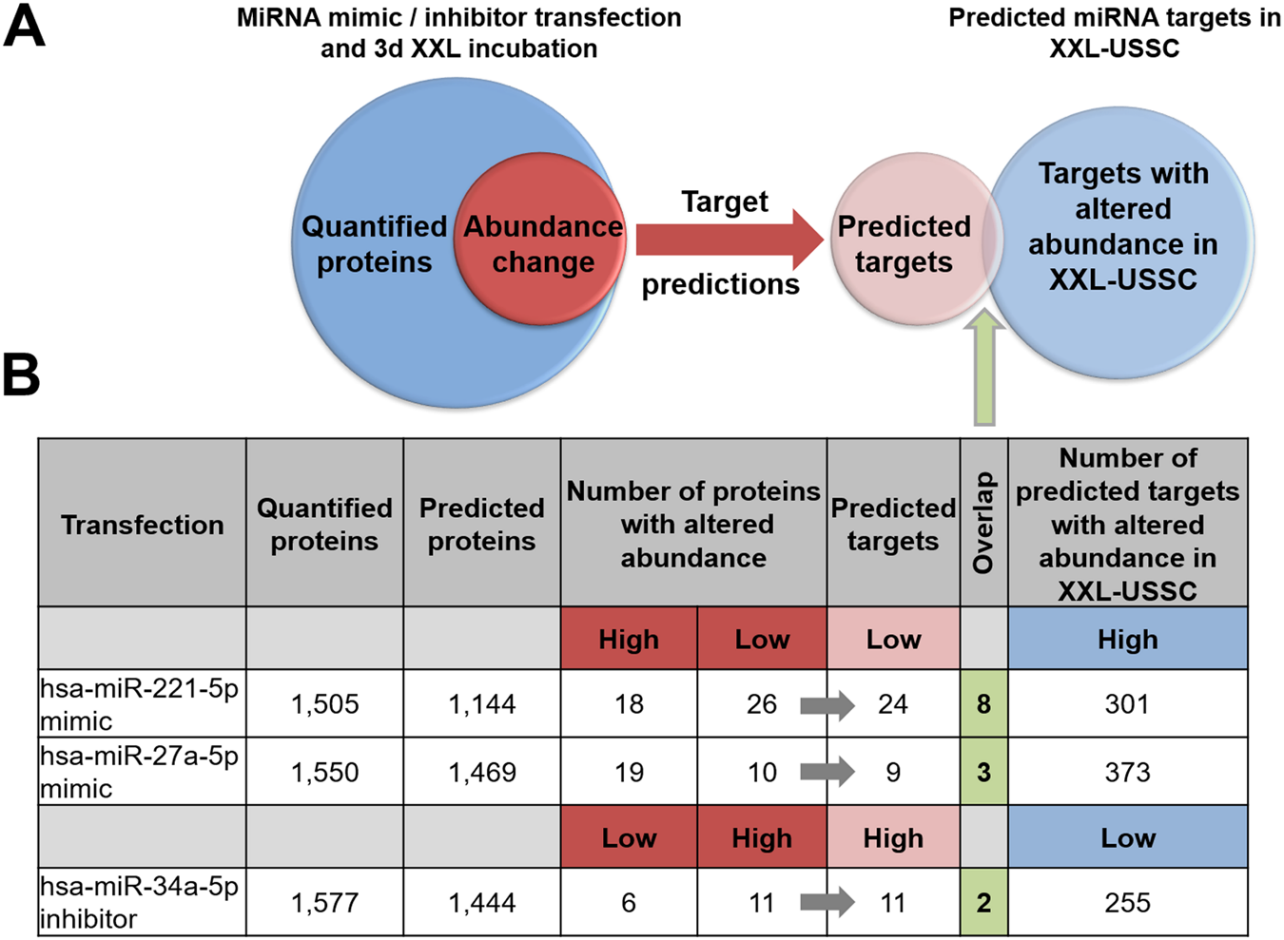
Summary of bioinformatic analysis of miRNA transfections and subsequent differential protein expression in XXL-USSC. (**A**) Bioinformatic workflow. Proteins regulated in 3d XXL-USSC upon transfection with mimics/inhibitors (left Venn diagram) were filtered for predicted targets (red arrow, using all 12 algorithms from miRWalk 2.0) and cross-analysed with inversely regulated predicted targets in non-transfected XXL-USSC (right Venn diagram). (**B**) For each miRNA mimic/inhibitor transfection, numbers of quantified proteins in transfected 3d XXL-USSC, predicted target proteins hereof, proteins with altered abundance (high and low) upon transfection, predicted targets hereof, and overlap (light green cell/column) with inversely regulated predicted targets (changes in abundance at 3d and/or 7d XXL incubation) in XXL-USSC are given.

We transfected USSC with miRNA mimics (hsa-miR-221-5p, hsa-miR-27-5p) or hairpin inhibitors (hsa-miR-34a-5p) 24 h prior to XXL-incubation followed by proteome analysis 3 days after XXL-induction in comparison to 3d XXL-USSC pre-transfected with an unspecific negative control (n.t. siRNA). Detailed proteome data are given in Supplementary Tables S11–S14. Transfection of both hsa-miR-221-5p and hsa-miR-27a-5p mimics led to a clear separation from the controls as revealed by PCA and volcano plots (Fig. 6A–D). A smaller segregation, however, was observed between hsa-miR-34a-5p hairpin inhibitor and control transfected cells (Fig. 6E,F). Subsequently, we analysed the overlap between regulated putative target proteins in transfected USSC and predicted target proteins inversely regulated in XXL-USSC (see workflow Fig. 7A).

We identified 1,505 and 1,550 proteins upon transfection of hsa-miR-221-5p and hsa-miR-27a-5p mimics from which 1,144 and 1,469 proteins were predicted targets according to aforementioned target gene predictions, respectively (Fig. 7B). Transfection of hsa-miR-34a-5p hairpin inhibitors resulted in the identification of 1,577 proteins including 1,444 predicted targets. The large portion of predicted targets among the identified proteins reflects the high numbers of predicted target proteins. Apparently, only a small portion of the identified predicted target proteins were indeed lower abundant upon transfection of hsa-miR-221-5p or hsa-miR-27-5p mimics in 3d XXL-USSC (24 proteins out of 1,144 predicted proteins and 9 regulated out of 1,469 predicted proteins, respectively, see Fig. 7B). The same was true for proteins higher abundant upon hsa-miR-34a-5p hairpin inhibitor transfection (6 proteins out of 1,444 predicted proteins, see Fig. 7B). These transfection responsive predicted target proteins likely represent true targets of the respective transfected miRNA.

In addition, we observed only a small overlap between predicted target proteins differentially abundant upon mimic or inhibitor transfection and those predicted target proteins that had been *inversely* regulated in non-transfected XXL-USSC (eight proteins out of 301 after hsa-miR-221-5p mimic transfection, three proteins out of 373 after hsa-miR-27-5p mimic transfection, two proteins out of 255 after hsa-miR-34a-5p hairpin inhibitor transfection; see Fig. 7B and Supplementary Table S14). It is noteworthy that several of these overlapping putative targets were predicted by only one single algorithm and RNAhybrid accounts for all these predictions (Supplementary Tables S9, S10, and S14). Only one overlapping protein (RDH10) was predicted by ≥5 algorithms and thus present in the virtual network (Fig. 5A).

Of note, mimic transfections also led to higher abundant proteins as well as vice versa upon hairpin inhibitor transfection (Fig. 7B), which is likely due to indirect effects rather than direct targeting by the respective miRNA.

In summary, our findings demonstrate that induction of USSC with XXL-medium is associated with significant changes in both, transcriptome and proteome with a good concordance between mRNA and protein regulation. For miRNAs, however, our experimental findings revealed (i) small numbers of miRNA transfection responsive targets under XXL conditions and (ii) a small overlap of these with inversely regulated proteins in non-transfected XXL-USSC, strongly indicating that miRNAs play only a minor role in shaping the proteome of XXL-USSC. Thus, miRNAs might act here as fine-tuning molecules rather than as primary regulators.

## Discussion

In the present study, we applied for the first time an integrative analysis of miRNome, transcriptome, and proteome together with functional miRNA analyses in order to estimate the impact of regulated miRNAs on the proteome of XXL-USSC. In an earlier quantitative PCR approach covering 377 miRNAs, we had demonstrated downregulation of various miRNAs, primarily from the miR-17-92 family, in XXL-USSC compared to native USSC^3^. Experimental target validations confirmed a bioinformatically predicted network between these miRNAs and several representative target proteins associated with neuronal development and function^3^. Although 3′-UTR-based sensor constructs demonstrated the capability of intracellular miRNAs to regulate these target genes in XXL-USSC^3^, no systemic data are yet available which estimate the *real* participation of differentially regulated miRNAs on the proteome in XXL-USSC. We thus employed USSC and XXL-derivatives as a model system to analyse the interplay between XXL-driven transcription and posttranscriptional miRNA-regulation a global level rather than investigating particular miRNA target gene interactions. As the current mirBase (release 22.1, October 2018) lists 2,693 mature human miRNAs, we now used NGS together with proteome analyses in order to get a complete picture of miRNA, mRNA, and proteome expression profiles and to include more detailed time kinetics.

The majority of up- and downregulation of mRNAs was observed already at 3d XXL (Fig. 1B,B’) implying that the switch to XXL leads to immediate changes in transcriptional patterns. It is worth noting that GO analysis of differentially regulated mRNAs at one hand points to cell cycle and proliferation-related genes (Fig. 1A), which is in line with the strong cell cycle arrest that occurs in USSC immediately upon addition of XXL. On the other hand, only few genes directly or indirectly related to neuronal function (neurofilament chains NEFL, NEFM and NEFH) or development (doublecortin, DCX) were upregulated even at 14d XXL demonstrating that XXL-USSC, despite having acquired a neuronal-like phenotype, have not differentiated into fully functional neurons within this period. It is worth noting that XXL-USSC show first phenotypic signs of neuronal lineage differentiation at 14d or later^27^ and since only a minority of cells are affected at these late time points, the resulting heterogeneity likely masks the analysis of neuronal pathways in these populations by NGS. Analysing neuronal pathways in XXL-USSC would require later time points which is compromised by the strong cells loss and was thus not the focus of our study.

In contrast to mRNAs, most of the miRNA regulation appeared at 7d and 14d of XXL-incubation (Fig. 4B,B’). In good agreement with our previous study^3^, miRNAs were predominantly downregulated at day 14. Since solely at 14d XXL far more mRNAs were upregulated rather than downregulated, this is at first glance in concordance with the inverse kind of regulatory pattern seen from miRNAs and implies that at this late time point the cells have achieved a new transcriptional balance.

Although apoptotic events prevent proteome analyses beyond 7d of XXL-incubation, comparison of full mRNA and proteome expression data revealed that a large majority of mRNAs and proteins were regulated in parallel (Fig. 3) with only few exceptions showing inverse regulation irrespective of statistical significance (Supplementary Fig. 3). Correlation between mRNA and protein regulation increased to 0.88 at both time points when only significantly regulated mRNAs were examined (Fig. 3D,D’). Comparison of significantly regulated mRNAs and significantly regulated proteins revealed that more proteins were solely up- or downregulated (Fig. 3B,B’). This is possibly due to proteins or mRNAs, which might still be regulated in parallel to their corresponding mRNA or protein, respectively, but only fail to meet our statistical criteria for differential expression.

In contrast to our observations in USSC and XXL-USSC, only a poor correlation of transcripts and proteins is seen in many biological systems^32–35^ although the interpretation of data might still be dependent on the underlying mathematical models^34^. Different correlations between transcriptomes and proteomes were observed in steady-state cells compared to cells undergoing biological transitions. In steady-state cells, transcripts seem to be the driving force behind the proteome^34,36^, whereas in state-transiting cells delays between mRNA regulation and subsequent proteome changes become visible within few hours^37,38^. Although induction with XXL also leads to USSC state transition, the far longer period of our analyses could not reflect these very early regulatory events. We observed an ongoing regulation of miRNAs, mRNAs and proteins in both regulatory directions over 3–7 days of XXL-incubation, with an emphasis on upregulation of mRNAs and proteins and downregulation of miRNAs (Figs. 1–4). It is likely that the proceeding upregulation of transcripts at 14d (Fig. 1B) also results in upregulation of corresponding proteins although cell loss prevents proteome analysis at this time point.

The overall inverse regulatory patterns of miRNAs and mRNAs/proteins in general could be expected from miRNA-mediated regulations and imply a network effect of miRNAs as we had proposed earlier^3^. The bioinformatic integration of regulated miRNAs and inversely regulated predicted target proteins indeed unfolded putative networks potentially participating in proteome shaping of XXL-USSC (Fig. 5). Bidirectional target gene redundancy of the regulated miRNAs is seen in both networks as several proteins are predicted to be regulatory targets of more than one miRNA. However, the estimation of the real *in vivo* contribution of these regulated miRNAs on the proteome is hampered by obviously unrealistically high numbers of predicted target genes using miRWalk 2.0 as described (Supplementary Tables S9 and S10) that mostly exceed the numbers of identified transcripts. As seen in the crossmatch tables in Supplementary Tables S9 and S10 only very few regulated proteins are, in fact, *no* predicted targets of the respective miRNAs. Removal of certain algorithms or limiting results to targets predicted by a (subjective) minimum number of algorithms as we did for the visualization of networks (minimum 5 out of 12, Fig. 5) might, however, result in a loss of true targets from the dataset. Therefore, even in view of the bioinformatic target predictions, it needs to be experimentally addressed whether the good correlation between mRNAs and proteins (Fig. 3D,D’) is solely achieved by mRNA transcription or to what extent it is further increased by inversely regulated miRNAs that influence protein abundance during XXL-incubation.

To analyse this balance, we linked miRNAs and proteome by transfecting USSC with selected miRNA mimics and hairpin inhibitors followed by 3d XXL-incubation and subsequent proteome analysis. Representing different regulatory directions and expression levels in USSC we used hsa-mir-27a-5p which is involved in cell cycle regulation and differentiation of pluripotent stem cells^39,40^ and hsa-mir-221-5p which is downregulated in RA-induced differentiation and also involved in cell cycle regulation^41,42^. In addition, we employed hsa-mir-34a-5p, known to be involved in tumour suppression, cell cycle arrest^43–45^, and regulation of neuronal differentiation^15,46^.

Our experimental approach of ectopic miRNA overexpression/inhibition cannot inherently discriminate between direct miRNA targeting and indirect miRNA effects. However, only small amounts (in total 44 out of 4057) of the quantified proteins predicted to be regulated by the respective miRNA showed an altered abundance in the expected inverse direction under conditions of ongoing XXL-incubation (Fig. 7B). It is thus likely that these 44 proteins are candidates for being *true* targets of the respective miRNA. However, in a more general view, our results also point to XXL as the major regulatory force in USSC able to counteract regulatory effects even from ectopically overexpressed or inhibited miRNAs. Remarkably, the predictions underlying the overall 44 transfection-responsive proteins (Fig. 7B) stem from the range of one single algorithm (RNA Hybrid, 13 proteins) up to 10 algorithms (1 protein) in parallel (Supplementary Table S14). This finding also demonstrates that possible true interactions can be predicted by just one single algorithm and reducing the number of predictions by restricting them to a certain minimum number of algorithms might indeed result in losses of possible true targets.

Proteomics is proposed to be a powerful tool to identify miRNAs and their regulatory targets and mechanisms^47^. In various studies, it has proven to be helpful to identify miRNA targets and regulation. Recently, stable isotope labelling with amino acids in cell culture (SILAC)-based mass spectrometry analysis helped to identify regulatory targets in miR-197-transfected rhabdomyosarcoma cells^48^. In addition, proteome, transcriptome and miRNome data identified networks related to cardiotoxicity in human pluripotent stem cells^49^. In this manner, miR-145 targets were identified in pancreatic cancer^50^ and several putative miRNA targets were found in intestinal cell lines^51^. Furthermore, a crosstalk between proteome and non-coding RNAs was proposed by proteome and miRNome data during ongoing endothelial senescence^52^ and during telomere shortening in cancer cells^53^. Possible targets for miR-23a, miR-24-2, and miR-27a were identified by mass spectrometry in pre B-lymphoblasts^54^. On proteome-wide scale, ectopic expression of hsa-mir-34a caused moderate changes in protein translation^44^. In glioma cells, overexpressing the brain-specific miR-128, 11 out of 13 selected candidate proteins derived from proteome data were validated by luciferase assays as potential targets^55^. Knock-down of miR-21 in breast cancer cells resulted in an abundance increase of 58 potential target proteins and 6 out of 12 candidates were validated successfully^56^.

With our USSC/XXL-USSC model system we did not primarily address the experimental identification of new miRNA targets in a global manner. The observation that ectopically overexpressed or inhibited miRNAs did not induce large changes in the proteome of XXL-USSC, however, does not devalue the virtual networks presented in Fig. 5, but in fact implies that XXL-driven mRNA transcription is the dominant regulatory force in proteome modelling, fully in line with the observed good correlation of mRNA and proteome in XXL-USSC. In this view, miRNAs likely act as more fine-tuning elements, supporting the cells to shape the primarily XXL-induced proteome. This is further strengthened by the finding that from the total of 44 predicted targets with altered abundancies upon transfection with miRNA mimics or inhibitors only 13 overlapped with the total of 929 predicted targets with reversely altered abundancies in non-transfected XXL-USSC (Fig. 7B) with only one of these (RDH10) predicted by ≥5 algorithms (Fig. 5A).

It must be noted that these results only reflect the impact of a single transfected miRNA. As several miRNAs are significantly regulated in parallel (Table 1), it is possible that our results underestimate the *in vivo* impact of several miRNAs regulated in parallel, especially in view of the miRNA target gene redundancy. This is supported by our earlier observation that 3′-target-UTR sensor constructs indeed report an influence of downregulated miRNA-populations in XXL-USSC upon however XXL-decoupling normalization^3^. In upcoming experiments, analyses of single miRNAs should therefore be broadened to a wider range of individual miRNA candidates and transfection of miRNA batches need to be considered for effective analysis of the coordinated impact of the miRNome on proteome regulation in a global manner.

Summarized, even upon ectopic overexpression or inhibition, the tested miRNAs induced only minor changes in the proteome of XXL-USSC. This indicates a dominant influence of XXL induced transcription and, on the other hand, more fine-tuning effects of the miRNA candidates in their normal *in vivo* expression situation rather than being primary regulatory tools.

## Methods

### Cell culture and cell differentiation

USSC were isolated from human umbilical cord blood as previously described by Kögler *et al*.^21^ and characterised by Hox-gene^23^ and delta-like 1 (DLK-1) expression^57^. USSC lines were provided by the Institute for Transplantation Diagnostics and Cell Therapeutics, Heinrich-Heine-University Medical Center, Düsseldorf, Germany. Informed consent was obtained from the donors’ mothers and has been approved by the Ethics Committee of Heinrich-Heine-University Düsseldorf Medical School. All experiments were performed in accordance with relevant guidelines and regulations (Ethics Committee of Heinrich-Heine-University Düsseldorf Medical School, approval number 3484). For both proteome and transcriptome analysis, USSC were cultured and differentiated in parallel as previously described with modifications^3,27,28^. In brief, USSC were expanded in Dulbecco’s modified Eagle’s medium (Lonza, Cologne, Germany) supplemented with 30% heat-inactivated FBS (Biochrom, Berlin, Germany), 2 mM glutamine (Gibco^TM^, Darmstadt, Germany) and penicillin/streptomycin (100 U/ml, Gibco^TM^) at 37 °C, 5% CO_2_ and 98% humidity. For analyses of proteome, transcriptome and miRNome, USSC lines 4/101, 4/146, 5/03, 5/73, 7/18, and 8/77 were cultured in parallel. USSC were seeded on poly-D-lysine (PDL, 0.5 mg/ml) and laminin (13 µg/ml) pre-coated glass coverslips in 6-well plates with a density of 110,000–140,000 USSC per well. 24 h later, USSC were incubated with differentiation medium (XXL)^27^ including DMEM GlutaMAX^TM^ (Gibco^TM^), 15% FBS, penicillin/streptomycin, 50 ng/ml beta-NGF, 20 ng/ml basic FGF (both Peprotech), 1 mM dibutyryl-cAMP, 0.5 mM 3-isobutyl-methylxanthine and 10 µM all-trans-retinoic acid (all Sigma-Aldrich, Steinheim, Germany). Medium was changed every 2–3 days. USSC were used in passage 7–9 for all experiments.

For quality control of the XXL-induction, at each time point analysed by LC-MS/MS as well as NGS (native, 3d XXL, 7d XXL, 14d XXL), immunocytochemical stainings were performed. USSC were fixed using 3.7% formaldehyde (Merck) for 15 min and carefully washed with PBS. Afterwards, fixed USSC were incubated with blocking solution containing 10% normal goat serum (Sigma Aldrich) and 0.03% Triton x-100 (Sigma Aldrich) for 1 h. To characterise USSC cultures at different stages, the neuronal marker neurofilament (mouse anti-neurofilament, Sigma Aldrich, MAB1592, 1:500 dilution in blocking solultion) and the proliferation marker Ki-67 (mouse anti-Ki-67, Millipore, MAB4190, 1:500 dilution in blocking buffer) were stained over night at 4 °C. After washing with PBS, secondary antibodies (goat anti-mouse Alexa 488 and goat anti-mouse Alexa 594, both Invitrogen, 1:500 dilution in PBS) were incubated for 3 h at room temperature. DAPI (4′,6-diamidino-2-phenylindole) staining was performed to label all cell nuclei. Coverslips were mounted in Fluoromount G^®^ (SouthernBiotech, Birmingham, USA). Images were taken using AxioPlan2 microscope (Zeiss).

### Sample preparation for proteome analysis

Native and XXL-USSC were washed three times with PBS to remove FBS and subsequently removed by scraping the cells with a cell scraper in 1 ml ice-cold PBS per well. Cell suspension was centrifuged at 800 × g for 5 min at 4 °C, the supernatant was removed, and cells were lysed by adding buffer containing 30 mM Tris Base, 7 M urea, 2 M thiourea, 4% CHAPS (pH 8.5). The lysate was sonicated 6 × 10 sec and centrifuged at 16,000 × g for 15 min. Protein concentration of the supernatant was determined by Pierce™ 660 nm Protein Assay (Thermo Fisher Scientific). For in-gel digestion, 5 µg protein per sample was loaded on an SDS-PAGE and run shortly for ~10 min (5 mm running distance). Afterwards, proteins were visualized by silver staining according to Nesterenko *et al*.^58^. The resulting lane was cut out, decolorized with a 1:1 mix of 30 mM sodium thiosulfate (Fluka) and 100 mM potassium hexacyanoferrate (III) (Merck), reduced with 10 mM dithiothreitol (Serva) and alkylated with 55 mM iodacetamide (Sigma Aldrich). Overnight trypsin digestion (2 µg, Serva) was performed at 37 °C and peptides were extracted with 50% acetonitrile (Sigma Aldrich) and 0.05% trifluoroacetic acid.

### LC-MS/MS

Extracted peptides were separated by a Ultimate 3000 RSCLnano System (Thermo Fisher Scientific) with a Acclaim PepMap100 trap column (3 µm C18 particle size, 100 Å pore size, 75 µm inner diameter, 2 cm length, Thermo Fisher Scientific) as a precolumn using 0.1% TFA as a mobile phase and a Acclaim PepMapRSLC (2 µm C18 particle size, 100 Å pore size, 75 µm inner diameter, 25 cm length, Thermo Fisher Scientific) as analytical column. The flow rate was constant with 300 nl/min using a 2 h gradient of 0.1%FA (Fluka) to 0.1% FA/60% acetonitrile. Separated peptides were eluted via nano electrospray ionization into the mass spectrometer (QExactive hybrid quadrupole-orbitrap mass spectrometer, Thermo Fisher Scientific). Mass spectra were recorded in positive ion mode with a mass range of 300–2,000 m/z and a resolution of 70,000. Up to ten precursors (+2, +3 charge states) were isolated within a 2 m/z isolation window and fragmented via higher-energy collisional dissociation. MS/MS spectra were recorded in centroid mode with a maximal ion time of 60 ms and a target value for the automatic gain control set to 100,000. The resolution was 17,500 at a scan range of 200 to 2,000 m/z. Already fragmented precursors were excluded from further isolation for the next 100 s.

### LC-MS/MS data analysis

Proteome Discoverer (Version 1.4, Thermo Fisher Scientific http://www.thermoscientific.com/en/product/proteome-discoverer-software.html) and Mascot search engine were used for protein identification. MS/MS spectra were searched against the UniProtKB Database (version: 2016/02). Following search parameters were used: Mass tolerance of 10 ppm (MSmode) and 0.4 Da (MS/MS mode), enzyme specificity was trypsin, two missed cleavage sites were considered during the search against human database. Mass range setting was 350–5,000 Da. Carbamidomethylation of cysteine was set as fixed modification. Oxidation of methionine was accepted as variable modification. For positive identification, we considered a false discovery rate (FDR) of <1% on peptide level (high peptide confidence, default p < 0.01). For FDR calculation, a decoy approach based on reversed protein sequences using Proteome Discoverer was applied. Label-free relative quantification was performed with Progenesis QI for proteomics 2.0 (Nonlinear Dynamics, Newcastle upon Tyne, http://www.nonlinear.com/progenesis). Only non-conflicting peptides were taken into account. Automatic alignment of runs to reference run was at least 84.3%. For filtering, peak picking limits were set to automatic and the maximum charge was set to 3. Normalization factor (NF) was ≤2 except one sample (NF 2.68). Peptides with a score <20 and a mass error >10 ppm were excluded from quantification. A minimum of 2 unique peptides per protein was required for quantification. R was used for statistical analysis calculating differences between samples by ANOVA (FDR corrected p-value or q-value <0.05) and Tukey’s post-hoc test (p < 0.01).

### Next generation sequencing

For sequencing, USSC were lysed in culture dish by adding 750 µl QIAzol (Qiagen, Hilden, Germany) after washing with PBS. RNA from native and XXL-USSC was isolated by miRNeasy Mini Kit (Qiagen). Quality and quantity of RNA samples were measured on a 2100 Bioanalyzer (Agilent, Amstelveen, the Netherlands). Integrity numbers ranged from 8.2 to 10. For RNA sequencing, a maximum of 1 µg total RNA was used for library preparation with TruSeq Small RNA Sample Prep Kit (#RS-200-0012/0024, Illumina, San Diego, CA). 300 ng of total RNA were used for library preparation with TruSeq Stranded Total RNA Sample Prep Kit with Ribo-Zero Gold (#RS-122-2301, Illumina). Size and DNA concentration of prepared cDNA libraries were also analysed on a 2100 Bioanalyzer. A concentration of seven picomolar of template DNA was loaded per flow cell and high-throughput sequencing was performed on the Illumina HiSeq. 2500 with 50 cycles (miRNAs) or 100 cycles (mRNAs).

### Sequencing data analysis

Sequencing reads for mRNA and miRNA sequencing were handled in a similar fashion. We obtained a total of 409,931,711 sequencing reads for mRNA-Seq and 273,704,169 sequencing reads for miRNA-Seq. First, adapter sequences and low quality ends were trimmed off using cutadapt^59^ and seqtk (https://github.com/lh3/seqtk). The remaining reads longer than 25/14 bases for the mRNA/miRNA dataset were then aligned against the human reference sequence GRCh38/hg38. We used STAR v2.4.0^60^ for the mRNA-Seq data and BWA v0.7.8a^61^ for the miRNA-Seq data. BWA was run with the -n 0.04 option to allow for errors in the short reads. A total of 165,833,130 and 65,820,517 were mapped uniquely to the genome for the mRNA and miRNA datasets, respectively. To estimate the expressions, reads were counted per gene annotated in Ensembl Genes V85^62^ and per miRNA annotated in miRBase V21^63^ using HTseq.^64^. Read counts were intra-sample normalized according to the sequencing depth of each sample with edgeR^65^, resulting in counts per million (CPM), which provided the basis for heatmap visualizations. To calculate differentially expressed genes and miRNAs, we have used edgeR functions glmQLFit and glmQLFTest. FDRs are reported which corrects for multiple testing. Only transcripts which are also listed as a protein in the UniProt database were taken into account to exclude non-coding transcripts (e.g. long non-coding RNAs) from further analysis. To exclude transcripts with extremely low CPM values of no biological significance, all sequencing data used for subsequent fold change analyses were filtered by CPM > 1 at least at one time point. Genes fulfilled significance criteria with p < 0.01, q < 0.05, FC > 1.5. In addition, miRNAs were selected from significantly regulated with p < 0.01, q < 0.05, FC > 1.5 at 3d, 7d and 14d XXL (same regulation direction).

### Bioinformatic analysis

Follow-up processing of the normalized transcriptome, microRNA and proteome data was done within the R/Bioconductor1 environment employing the packages gplots 2 and heatmap.plus^66,67^. Cluster analysis and heatmaps were calculated with the heatmap.2 and heatmap.plus functions from these packages. Genes, microRNAs and proteins were included in the heatmap when passing a threshold for the coefficient of variation. Colours were scaled for each row corresponding to a gene, microRNA or protein. Pearson’s correlation was used as similarity measure.

MiRNA target gene predictions were performed using the miRWalk 2.0 website (http://zmf.umm.uni-heidelberg.de/apps/zmf/mirwalk2/index.html)^30^ combining 12 individual algorithms (miRWalk, miRDB, PITA, MicroT4, miRMap, RNA22, miRanda, miRNAMap, RNAhybrid, miRBridge, PicTar2, and Targetscan) using the default setting except for the pValue, which was changed from 0.05 to 0.01 (and appears to affect miRWalk-results only). To increase the reliability of predictions, we only included putative targets predicted by ≧5 individual algorithms. The resulting gene lists were matched to the NGS data on the level of the respective Ensembl IDs. Gene lists from proteome data, NGS data and target predictions were analysed for gene ontology by using the DAVID database (https://david.ncifcrf.gov/)^68,69^. Scatter plots, principle component analyses and correlations were calculated by using the software package Perseus^70^. Calculation of Venn diagrams was performed with FunRich^71^. Protein-miRNA networks were visualised by using Cytoscape 3.7.1^72^.

### Transfection of USSC

For transfection experiments, USSC line 5/03 was used. USSC were seeded on PDL and laminin pre-coated glass coverslips with a density of 150,000-170,000 USSC/well on 6 well plates and each well was transfected with 3 pMol of the respective miRIDIAN miRNA mimic or inhibitor (Dharmacon) using Dharmafect (Dharmacon) according to the manufacturer’s protocol. USSC were incubated with XXL-medium 24 h after transfection and proteomes were analysed 3 days upon XXL-induction as described above.

## Supplementary information

Supplementary Figures 1–3 and Legends to Supplementary Tables.

Supplementary Table S1-S2-S3.

Supplementary Table S4-S5-S6.

Supplementary Table S7-S8.

Supplementary Table S9.

Supplementary Table S10.

Supplementary Table S11-S12-S13-S14.

## Data Availability

All the data analysed during this study are included in this published article (and its Supplementary Information files). We confirm that all the data in this manuscript is original. The mass spectrometry proteomics data have been deposited to the ProteomeXchange Consortium (http://proteomecentral.proteomexchange.org) via the PRIDE partner repository^73^ with the dataset identifier PXD016915. In addition, next generation sequencing data discussed in this publication have been deposited in NCBI’s Gene Expression Omnibus^74^ and are accessible through GEO Series accession number GSE144466.
