# Supplementary material for: Functional omics analyses reveal only minor effects of microRNAs on human somatic stem cell differentiation
Source: Sci Rep. 2020 Feb 24;10:3284. doi: 10.1038/s41598-020-60065-8 (PMC7040006; doi:10.1038/s41598-020-60065-8)
Supplement: Supplementary file 1 — Supplementary Figures 1–3 and Legends to Supplementary Tables. [file 41598_2020_60065_MOESM1_ESM.pdf]

Functional omics analyses reveal only minor effects of microRNAs on human somatic stem cell differentiation

Authors: Jessica Schira-Heinen, Agathe Czapla, Marion Hendricks, Andreas Kloetgen, Wasco Wruck, James Adjaye, Gesine Kögler, Hans Werner Müller, Kai Stühler, Hans-Ingo Trompeter

Supplementary Figures

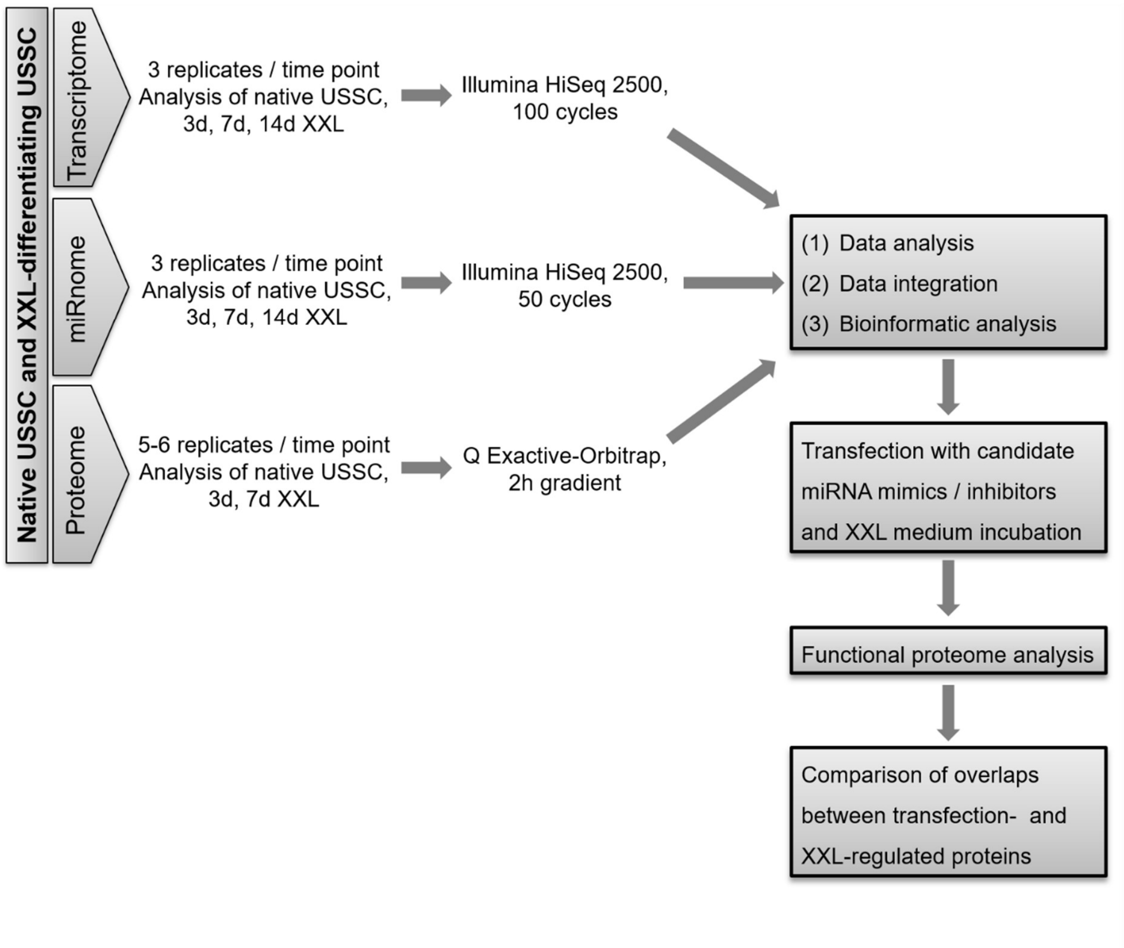

Supplementary Fig. 1. Experimental workflow.

This scheme depicts the experimental workflow of the presented study.

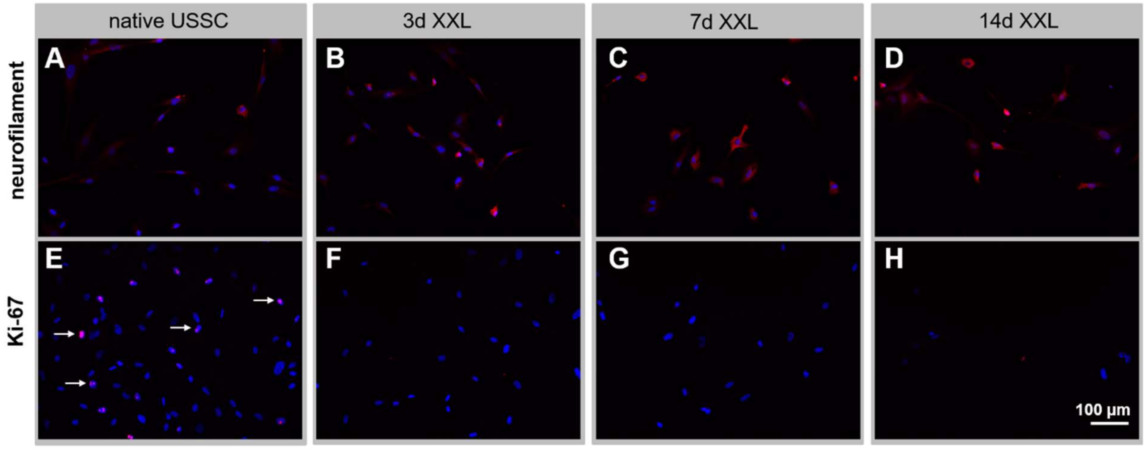

Supplementary Fig. 2. Quality control of XXL-USSC.

Representative photographs of native USSC as well as of 3d, 7d, and 14d XXL-USSC are shown. Upper row: stainings for neurofilament and DAPI, lower row: stainings for proliferation marker Ki-67 and DAPI. Arrows denote Ki-67 positive cells. Note the ongoing apoptotic loss of cells during XXL-induction.

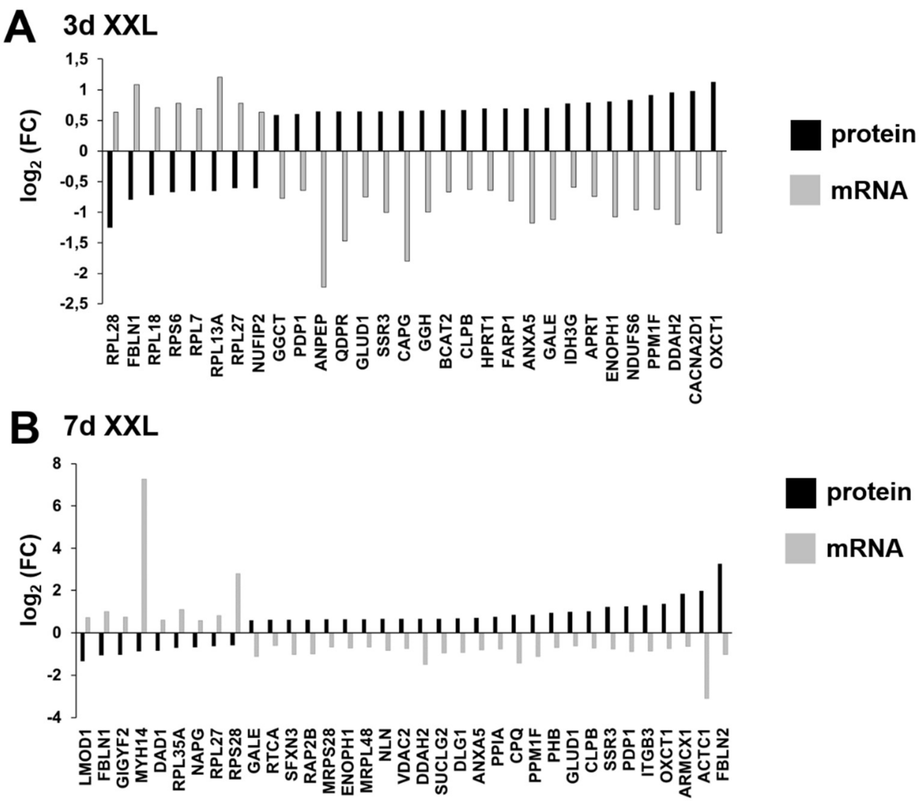

Supplementary Fig. 3. Inverse regulation of corresponding genes and proteins in XXL-USSC.

Proteins and corresponding mRNAs which have an inverse regulation pattern (FC > 1.5) at time points 3d (**A**) and 7d (**B**) of XXL incubation are shown. Here, also proteins and mRNAs are included which do not meet the significance criteria.

Legends for Supplementary Tables

Supplementary Table S1-S2-S3. Quantified mRNAs in USSC and XXL-USSC and cluster-specific gene ontology (GO) term analysis.

Table S1: Transcriptome of native and XXL-USSC. Single CPM values are included for each biological replicate (columns C-N).

Table S2: Quantitative transcriptome data showed an overlap of 1797 proteins which have been quantified by label-free mass spectrometry approach (accession numbers, column D). Mean CPM (columns E-H), log_2FC, p-values and q-values are listed. Native USSC are compared to XXL incubated USSC for each analysed time point (columns I-Q)).

Table S3: Cluster-specific gene ontology (GO) term analysis. Two clusters derived from cluster analysis (see Fig. 1C) were analysed including 746 and 857 mRNAs, respectively (column A and B). Overview of all biological processes (column C) found to be enriched by DAVID database with FDR corrected p-value ($q < 0.05$). In addition, numbers of transcripts (column D) and percentage of transcripts from total transcripts included in the depicted biological process (column E) are listed.

Supplementary Table S4-S5-S6. Quantified proteins in USSC and XXL-USSC and cluster-specific gene ontology (GO) term analysis.

Table S4: Quantitative comparison of native, 3d XXL and 7d XXL incubated USSC by label-free mass spectrometry approach. Only proteins identified with at least 2 unique peptides (column E) and a score of at least 20 (column F) are included (1864 proteins). Label-free quantification (LFQ) intensities of all replicates are listed (columns G-W).

Table S5: Overview of mean LFQ intensities from biological replicates for each analysed time point (columns G-I). Fold changes (log_2FC, columns K, M, O), q-value (column P) of < 0.05 and p-value (columns Q-S) of < 0.01 after pairwise comparison were considered for significantly changed abundances.

Table S6: Cluster-specific gene ontology (GO) term analysis. Two clusters derived from cluster analysis (see Fig. 2B) were analysed including 391 and 381 proteins, respectively (column A and B). Overview of all biological processes (column C) found

to be enriched by DAVID database with FDR corrected p-value ($q < 0.05$). In addition, numbers of proteins (column D) and percentage of proteins from total proteins included in the depicted biological process (column E) are listed.

Supplementary Table S7-S8. Quantified miRNAs in native USSC and XXL-USSC.

Table S7: Overview of CPM values from 592 analysed miRNAs after filtering by CPM > 1 at least at one time point in three USSC lines (4/101, 4/146, and 5/03) each at native stage, and at 3d, 7d and 14d of XXL incubation.

Table S8: Overview of mean CPM values from native USSC and after 3d, 7d, and 14d of XXL incubation. Log_2 fold changes and corresponding p- and q-values at 3d XXL, 7d XXL and 14d XXL each compared to native USSC are given. Lines 2-14 (green): miRNAs significantly downregulated (red) in 14d XXL-USSC (see Table 1); lines 15-29 (red): miRNAs significantly upregulated in 14d XXL USSC (see Table 1); lines 30-593: remaining miRNAs not further discussed.

Supplementary Tables S9 and S10. Bioinformatic target gene predictions of significantly regulated miRNAs.

Summary of bioinformatic target gene predictions using miRWalk 2.0 including 12 algorithms for hsa-miR-221-5p, hsa-miR-24-2-5p, hsa-miR-27a-5p, hsa-miR-222-5p, and hsa-miR-138-1-3p (downregulated, Supplementary Table S9), hsa-miR-146a-5p, hsa-miR-34a-5p, and hsa-miR-212-5p (upregulated, Supplementary Table S10), each on individual sheets.

Herein, predictions from each algorithm are given separately (columns F-Q), together with the sum of algorithms predicting a given target (column R). Of note, the column "Gene name" (official gene symbols) contains redundancies due to several RefSeq IDs often matching a single gene (i.e. transcription variants). Column S in both files summarises all predicted genes from column C after removal of redundancies.

The sheets "Crossmatch..." in both files present crosstables of regulated miRNAs and inversely regulated predicted target proteins. Regulated proteins in XXL-USSC (3d and/or 7d) are listed in columns A (3d XXL) and/or B (7d XXL) separately, sorted by

the number of predicting miRNAs. Predictions from all 12 algorithms of miRWalk 2.0 are used. The subsets of predictions from at least 5 algorithms were also used for network constructions (**Fig. 5**).

Supplementary Table S11-S12-S13-S14. Quantitative proteome analysis after miRNA mimic or hairpin inhibitor transfections.

Table S11: Label-free quantitative proteome analysis of control (n.t. siRNA) and hsa-miR27a-5p mimic transfected USSC and subsequent XXL incubation for 3 days. Only proteins identified with at least 2 unique peptides (column E) and a score of at least 20 (column F) are included (1,550 proteins). Proteins which are predicted targets for hsa-miR-27a-5p (1,469 proteins) as well as the number of prediction algorithms are listed (column G and H). In addition, label-free quantification intensities of all replicates are shown (columns I-Q). Fold changes (FC >1.5) and a q-value of < 0.05 are considered for significantly changed abundances.

Table S12: Analysis as described for Table S10 but using hsa-miR-221-5p mimic. Here, 1,505 proteins are included (column F); 1,144 were predicted hsa-miR-221-5p targets (columns G and H).

Table S13: Analysis as described for Table S10 but using hsa-miR-34a-5p inhibitor. Here, 1,577 proteins are included (column F); 1,444 were predicted hsa-miR-34a-5p targets (columns G and H).

Table S14: Summary the predicted target proteins significantly (q < 0.05, FC > 1.5) lower or higher abundant upon miRNA mimic or hairpin inhibitor transfection (see **Fig. 7**) together with the number of algorithms (out of 12) predicting the particular protein. Of note, all one-algorithm predictions stem from algorithm RNAhybrid (bold numbers). Proteins in bold green are inversely regulated in XXL-USSC vs. USSC (see **Fig. 7**).
